# Self-pollination rate and floral-display size in *Asclepias syriaca* (Common Milkweed) with regard to floral-visitor taxa

**DOI:** 10.1186/1471-2148-14-144

**Published:** 2014-06-23

**Authors:** Aaron F Howard, Edward M Barrows

**Affiliations:** 1 Department of Biology, Georgetown University, Box 571229, Washington, DC 20057-1229, USA; 2 Center for the Environment, Georgetown University, Box 571229, Washington, DC 20057-1229, USA

**Keywords:** *Apis mellifera*, Apocynaceae, *Bombus*, Insect pollinators, *Asclepias syriaca*, Floral-display size, Geitonogamy, Mating system, Self-pollination rate

## Abstract

**Background:**

Animals fertilize thousands of angiosperm species whose floral-display sizes can significantly influence pollinator behavior and plant reproductive success. Many studies have measured the interactions among pollinator behavior, floral-display size, and plant reproductive success, but few studies have been able to separate the effects of pollinator behavior and post-pollination processes on angiosperm sexual reproduction. In this study, we utilized the highly self-incompatible pollinium-pollination system of *Asclepias syriaca* (Common Milkweed) to quantify how insect visitors influenced male reproductive success measured as pollen removal, female reproductive success measured as pollen deposition, and self-pollination rate. We also determined how floral-display size impacts both visitor behavior and self-pollination rate.

**Results:**

Four insect taxonomic orders visited *A. syriaca*: Coleoptera, Diptera, Hymenoptera, and Lepidoptera*.* We focused on three groups of visitor taxa within two orders (Hymenoptera and Lepidoptera) with sample sizes large enough for quantitative analysis: *Apis mellifera* (Western Honey Bee), *Bombus* spp. (bumble bees) and lepidopterans (butterflies and moths). Qualitatively, lepidopterans had the highest pollinator importance values, but the large variability in the lepidopteran data precluded meaningful interpretation of much of their behavior. The introduced *A. mellifera* was the most effective and most important diurnal pollinator with regard to both pollen removal and pollen deposition. However, when considering the self-incompatibility of *A. syriaca*, *A. mellifera* was not the most important pollinator because of its high self-pollination rate as compared to *Bombus* spp. Additionally, the rate of self-pollination increased more rapidly with the number of flowers per inflorescence in *A. mellifera* than in the native *Bombus* spp.

**Conclusions:**

*Apis mellifera*’s high rate of self-pollination may have significant negative effects on both male and female reproductive successes in *A. syriaca*, causing different selection on floral-display size than native pollinators.

## Background

In most angiosperm species, fertilization depends on pollinating vectors, such as air-currents and animals. Floral characteristics, including color, display architecture, phenology, scent, shape, size, and spatial arrangement, influence animal-pollinator behavior, which in turn impacts the deposition of self- and outcross pollen on stigmas [1]. The magnitudes of animal-pollinator effects on plant reproductive success have been estimated in several ways including (1) the proportion of visitors bearing pollen [2]; (2) the rate of pollen deposition, including both self- and outcross pollen [3]; (3) the amount of pollen removed from anthers [4]; (4) the degree of fruit and seed set [5,6]; (5) the rate of geitonogamy (intra-plant pollen deposition on stigmas) [7]; and (6) the proportion of seeds produced as a result of outcrossed versus self-pollen deposition [8,9]. However, previous studies (except [10]) that quantified pollen movement alone, lacked estimates of genetic transmission through pollination, and the studies that measured fruit and seed production alone could not differentiate between possible effects of cross- and self-pollen deposition and post-pollination incompatibility processes.

Many of the above studies found a relationship between pollinators and plant reproductive success, as well as a statistical interaction among pollinator behavior, plant reproduction, and floral-display size (*e.g*., [8,9]). Pollinators often increase their rates of outcross-pollen deposition as plant population density, plant population size, or both increase [11,12]. This may result from increased pollinator visitation that decreases pollen limitation and intraplant, intrafloral autogamy (pollination of a flower with its own pollen) or a reduction in the number of flowers visited in succession on a plant, which reduces geitonogamy (pollination of a flower with pollen from another intraplant flower) [13-15]. Nonetheless, geitonogamy may also increase with floral-display size if pollinators increase the number of flowers that they visit on individual plants [9,13,16]. With regard to the pollination of a partly or entirely self-incompatible plant species, floral-display size can be a compromise between the size that attracts an adequate number of pollinators with outcross pollen and the size that causes pollinators to visit too many flowers of the same plant, which can result in increased self-pollen deposition [13]. Increased self-pollination through geitonogamy may result in reduced fecundity through male reproduction (seed siring) because of pollen discounting (*i.e*., reduced pollen transfer between plants) [9] and because self-pollen cannot sire many seeds (*e.g*., [13]).

Pollination and floral-display size have been studied frequently in *Asclepias* species because they have derived, pollinium-pollination systems conducive for investigating pollen movement. *Asclepias* pollen occurs in discrete sac-like structures called pollinia, which are the units of pollen dispersal [17]. Pollination does not occur unless an insect removes a pollinium from one flower and correctly inserts it into another, or the same, flower’s stigmatic slit. An *Asclepias* flower has five pollinaria, each comprising a corpusculum, two translator arms, and two pollinia. This pollinium morphology allows one to quantify discrete pollination events easily by macroscopic examination. Previous studies have shown that different pollinator types insert pollinia at different rates [7,18], and that floral-display size impacts reproductive success [1,19,20]. However, the influence of each level of floral display (*e.g*., an individual inflorescence, a stem with one or more inflorescences, or whole plant with one or more stems) on reproductive success and at which level selection on floral-display occurs is still in question [1,21-23].

As a group, North American *Asclepias* spp. are pollinated by a wide range of insects including native bees and the introduced *Apis mellifera* (Western Honey Bee), beetles, butterflies, flies, and moths [7,17,18,24-26]. *Apis mellifera* is an efficient pollinator of many plant species including several *Asclepias* spp. [7,18,27], but a few studies have demonstrated that this bee may detrimentally affect seed set in some plant species [28,29]. This may occur because on average *A. mellifera* moves among plants less than some native pollinators, even though it removes more pollen per visit [30,31]. Therefore, while *A. mellifera* may differ from native pollinators in visitation behavior [30-32] and is efficient at removing and depositing the pollen of some plant species, further studies are needed to determine its potential influence on plant reproductive success.

The majority of studied *Asclepias* spp. are primarily or completely self-incompatible [24]. This can potentially reduce the reproductive success of these species because, in addition to the negative consequences of pollen discounting, each *Asclepias* flower has only five stigmatic slits and self-pollen can clog these slits and compete with outcrossed pollen for ovules. *Asclepias* spp. have late-acting self-incompatibility systems, and their self-pollen tubes germinate and grow just as fast as outcross-pollen ones, which can reduce the number of compatible fertilizations [33]. However, estimating self-pollination rate is methodologically difficult, and only a few studies have made population-wide estimates for *Asclepias* spp. [34-36]. Additionally, while several studies have investigated the relative quantity of *Asclepias* pollen deposited by different pollinators [7,18], none have determined the source (intraplant or interplant) of the pollen, which is critical when quantifying reproduction in self-incompatible plants. Many studies have utilized non-genetic methods to measure pollen movement in other genera (*e.g*., [37-39]), and Matsuki et al. [40] have developed a methodology to genotype single pollen grains, but, to our knowledge, no published study has used genetic techniques to quantify self-pollination rate (as opposed to the rate of self-seed production) with regard to multiple floral-visitor taxa and floral-display size.

In this study, we utilized the *Asclepias* pollinium-pollination system to examine how the floral-display size of *Asclepias syriaca* (Common Milkweed) influences the visitation behavior of *A. mellifera* and native flower visitors and how visitor behavior influences the reproductive success of *A. syriaca*. Specifically, we tested three hypotheses: (1) different visitor taxa of *A. syriaca* behave differently when visiting its flowers; (2) the visitors’ behavioral differences relate to floral-display size; and (3) the visitors’ differing behaviors affect self-pollination rates. To test these hypotheses, we sampled two *A. syriaca* populations over 2 yr, and estimated pollen-deposition and the self-pollination rates for three insect-visitor taxa*.* Then we examined our estimates with regard to visitor behavior and the number of flowers per inflorescence and stem.

## Methods

### Study plant

*Asclepias syriaca* is a North American, clonal, perennial herb with one through several thousand stems (ramets) per plant (genet) [41]. This species usually grows in disturbed habitats, forest edges, roadsides, and open fields [42]. It is < 5% self-compatible [43,44]. Its long-lived flowers last a mean of approximately 5 days and remain in anthesis as long as 7 days [45]. Flowers of this species occur in umbellate cymes (a type of inflorescence) [46] with markedly different numbers of flowers per cyme and cymes per stem [42]. Because we could not differentiate between the ortets and their vegetatively propagated ramets in our study clones, we call all of them “stems” in this paper.

### Study sites

The study populations grew in a 30-km^2^ meadow at Woodend Nature Sanctuary in Chevy Chase, Maryland (39°3'N, 77°4'W) and in a managed 40-km^2^ prairie consisting of mostly cool-season grasses at Blandy Experimental Farm of the University of Virginia in Boyce, Virginia (39°3'N, 78°3'W). In addition to *A. syriaca*, Blandy Experimental Farm had several hundred *Asclepias incarnata* stems and a few *Asclepias tuberosa* stems. Woodend Nature Sanctuary had no other *Asclepias* species. We could find no information on the ages of our clones or how long their ortets live. We had permission to collect data at Woodend Nature Sanctuary and Blandy Experimental Farm in 2008 and 2009. Licenses were not required at either site.

### Field observations and measurements

To exclude insect visitors from inflorescences, we bagged one inflorescence per flowering stem 1–3 days before its anthesis began in June–July 2008 and 2009. We used bridal veil because it has the smallest effect on inflorescence microenvironment, nectar production, and visitor behavior compared to other netting materials ([47], pers. obs.). However, insects occasionally deposited pollinia through the bridal-veil bags, so we accounted for such insertions by collecting bagged inflorescences that never had their bags removed (control inflorescences). We measured the number of flowers per focal inflorescence (inflorescence size), the number of flowers per stem (stem size), stem height, and the number of inflorescences per stem.

First, immediately after collecting a stem’s floral-display data, we removed a focal inflorescence’s bag, observed the inflorescence’s first insect visitor, and recorded its behavior including the number of flowers visited on the focal inflorescence (called “the number of flowers visited”), number of inflorescences it visited, and its visiting time. Second, we attempted to collect each visitor after its visiting bout, but this was often not possible, especially with fast-flying lepidopterans. Therefore, we identified the 244 collected visitors to the lowest possible taxonomic level in our lab, and the rest of the visitors to the lowest taxonomic level possible in the field. Third, we removed the inflorescence from its stem in order to quantify the number of pollinia inserted and removed. Fourth, in the lab, we scored pollinium insertions and removals by examining each flower’s stigmatic chambers for inserted pollinia and anther regions for removed pollinia using a dissecting microscope at 10–30 magnification power [7,18]. Fifth, we removed inserted pollinia from the inflorescence’s stigmatic chambers and immediately placed them on ice for subsequent genetic analysis.

### Plant and pollinium genotyping

We genotyped maternal plants and inserted pollinia with up to four polymorphic-microsatellite-locus primer sequences from O’Quinn and Fishbein [48] (Asyr-C4, Asyr-C102, Asyr-C103, Asyr-C109). Maternal DNA was extracted from stem tissue collected with the focal inflorescences using the QIAGEN DNeasy Plant Mini Kit protocol (QIAGEN). Because pollinia are small, we extracted their DNA using a modified QIAGEN DNeasy Plant Mini Kit protocol where we lowered all of the reagent volumes by one order of magnitude and skipped the protein removal steps. Each microsatellite primer was fluorescently labeled (6-FAM, VIC, NED, and PET), and the maternal and pollinium genotypes were determined using an ABI PRISM 3100 Genetic Analyzer.

### Self-pollination rate

From the genotypic data, we estimated the self-pollination rates for each of the three visitor taxa. Direct estimation of self-pollination rates is the most powerful method for determining a plant’s fraction of self-pollen deposition. To estimate the direct self-pollination rate, we compared the multilocus genotype of a pollinator-inserted pollinium to the genotype of the visited inflorescence. If any of the pollinium alleles were different than the inflorescence’s alleles, we classified the pollinium as an outcrossed one. There is a finite probability that multiple plants have the same multilocus genotypes, through chance or relatedness, which could result in erroneously assigned insertions when the inflorescence and pollinium have the same genotype. Therefore, the direct-estimation method (*S*_d_) results in an unbiased estimate of the self-pollination rate only if the probability of erroneously assigned insertions is very small. Since each pollinium is an aggregate of haploid pollen grains from the same pollen source, we could genotype a pollinium to determine its producer’s diploid genotype [36]. This determination increased our probability of excluding potentially erroneous pollen sources and increased the power of our self-pollination rate estimation.

To control for possible erroneous pollen-source assignment, we quantified self-pollination rates using a method-of-moments estimator (based on [49] and [36] and modified from [50]), which we designated as *S*_*m*_. Typically, the probability of erroneous pollen source assignment (α) is estimated using pollen-pool allele frequencies from seeds [50], but in our study we used pollen-pool genotype frequencies [36]. Estimating the genotype frequencies in *A. syriaca* is potentially difficult because its clones can have up through thousands of stems per plant [42]. Considering each flowering stem to be a separate plant may result in a severe underestimation of the genotypic diversity in a population, but conversely assuming that each genotype is an individual plant may result in an overestimation of the frequency of rare alleles. Both of these biases may result in inaccurate allele-frequency estimation and a consequent inaccurate estimation of self-pollination rate. Therefore, to reduce any overestimation of rare alleles, we used a round-robin method of allele frequency estimation [51].

We were unable to estimate method-of-moments self-pollination rates with regard to each unique maternal genotype (*i.e.*, rate of self-pollinia inserted into flowers of inflorescences with the same genotype) because the median- and mode-inflorescence insertion rates were 0, so we estimated *S*_*m*_ for each visitor taxon (*S*_*mp*_). Direct α (α_*d*_) is

(1) $$\alpha_{d}=\sum_{i=1}^{N}w_{i}\left[\prod_{k=1}^{n}P_{\mathrm{ik}}\right],$$

where *P*_*ik*_ is the diploid genotype frequency of multilocus paternal genotype *i* at locus *k*, and *w*_*i*_ is the weighted frequency of inserted pollinia in inflorescences visited by a particular visitor taxon with multilocus pollinium genotype *i. N* is the number of unique maternal-diploid genotypes visited by a particular visitor taxon, and *n* is the number of loci. To control for potential bias in the self-pollination rate estimates due to insertions through the bags prior to visitor visitation, we used weighted-means estimates of α_*d*_ and the observed self-pollination rate to attribute the portion of both variables to bagged- (α_d*c*_ and *S*_*dc*_) versus visitor-inserted (α_*dp*_ and *S*_*dp*_) pollinia. The direct α for visitor-inserted pollinia (α_*dp*_) is

(2) $$\alpha_{\mathrm{dp}}=\frac{\alpha_{\mathrm{dT}}-\left(1-r_{p}\right)\alpha_{\mathrm{dc}}}{r_{p}}\;\mathrm{for}\;\alpha_{\mathrm{dT}}>\alpha_{c}\left(1-r_{p}\right),$$

where α_*dT*_ is the estimate of direct α for all insertions (both insertions through the bags and insertions into flowers of unbagged focal inflorescences), and *r*_*p*_ is the proportion of insertions attributed to each visitor taxon. The direct self-pollination rate for visitor-inserted pollinia (*S*_*dp*_) is similarly calculated as

(3) $$S_{\mathrm{dp}}=1-\left(\frac{t_{\mathrm{dT}}-\left(1-r_{p}\right)t_{\mathrm{dc}}}{r_{p}}\right)\;\mathrm{for}\;t_{\mathrm{dT}}>t_{\mathrm{dc}}\left(1-r_{p}\right),$$

where *t*_*dT*_ is the total direct-outcrossing rate, and *t*_*dc*_ is the direct-outcrossing rate for pollinia inserted into control inflorescences. We used the estimates of α_*dp*_ and *S*_*dp*_ to estimate the self-pollination rates (*S*_*mp*_) attributed to each visitor taxon using a method-of-moments equation [50].

When quantifying the relationship between self-pollination and floral-display size, we used the direct-estimation method of the self-pollination rate (*S*_*dp*_) for each maternal genotype again because we were unable to estimate method-of-moments self-pollination rates. We used eq. (3) to estimate *S*_*dp*_ for each maternal genotype, where *t*_*dT*_ is the total direct-outcrossing rate across all inflorescences that share the same genotype. We averaged floral-display size values across inflorescences with the same genotype.

### Pollinator effectiveness and importance

In order to evaluate the contribution of different floral visitors to pollen deposition and removal and accurately quantify their roles in plant reproductive success, it is important to incorporate other aspects of visitor-specific pollen movement with self-pollination rate, including pollen-deposition and pollen-removal rates and relative visitor abundance [52]. Therefore, we quantified pollinator effectiveness for both female reproductive success (measured as pollinium insertions) and male reproductive success (pollinium removals) per focal-inflorescence flower per visitor taxon, and pollinator importance, measured as the pollinator effectiveness multiplied by the relative abundance of each visitor taxon that deposited or removed pollinia. We estimated taxon relative abundance as the number of visits made by the focal visitor taxon divided by the total number of visits by *A. mellifera*, *Bombus* spp., and lepidopterans observed during our study period [52]. Additionally, since *A. syriaca* may be over 95% self-incompatible and only a small percentage (<5%) of self-pollinia insertions contribute to reproductive success [24], we generated another metric of pollinator importance that includes only the expected proportion of insertions that lead to seed production or siring (pollinia not lost to pollen discounting) that we term “self-incompatibility-controlled pollinator importance” (*SICPI*), which for insertions is calculated as

(4) $$\mathrm{SICP}I_{I}=PI_{I}\left(1-\left(S_{\mathrm{mp}}\mathrm{SI}\right)\right),$$

where *SI* is the rate of self-incompatibility, and *PI*_*I*_ is the pollinator importance for insertions. Self-incompatibility-controlled pollinator importance for removals is calculated as

(5) $$\mathrm{SICP}I_{R}=PI_{R}-\left(PI_{I}S_{\mathrm{mp}}\mathrm{SI}\right),$$

where *PI*_*R*_ is the pollinator importance for removals. We used a conservative estimate of 5% for self-compatibility reported in the literature [24] and which was also observed from hand-pollination experiments in our study populations (pers. obs.). We did not measure pair-wise self-incompatibility for all genotypes in the population, which would have allowed us to incorporate the variability of self-incompatibility into *SICPI.*

### Data analysis

We used a negative-binomial generalized linear model (GLM) to determine the statistical relationship between floral-display size and visitor behavior because of overdispersion and a quadratic mean-variance relationship in the number of flowers visited [53]. We used only the number of flowers visited in the behavior model because flowers visited and time visited were significantly correlated (r = 0.71, *P* < 0.01) and including both did not improve the explanatory power of our model. We used zero-inflated Poisson GLMs to determine whether floral-display size was a statistical predictor of pollinium-insertion and -removal rates per visit because a larger portion of the visits resulted in zero pollinium insertions or removals than would be predicted by a Poisson distribution [54]. The zero-inflated Poisson GLMs had two components, one that followed a Poisson distribution and another that followed a binomial distribution. The Poisson portion of the model included parameters for visitor taxon and floral-display size. The binomial portion included a parameter for visitor taxon and modeled the excess zeros that were not consistent with the expectations of a Poisson distribution. We included only a visitor taxon parameter in the binomial portion of the model because it predicted the number of observed zeros better than a model with just an intercept, and models with more parameters (*e.g*., inflorescence and stem sizes) did not improve goodness of fit, according to likelihood ratio tests.

We investigated the relationship between *S*_*dp*_, visitor taxon, and floral-display size using a quasi-binomial generalized linear-mixed model (GLMM) that included maternal genotype and site as random variables to control for visitors that visited the same maternal genotype more than once and because some genotypes, based on our markers, were found at both sites. Stem height and the number of inflorescences were initially included as measures of floral-display size, but were removed from all models because they had no explanatory power for our dependent variables. To estimate the variance in *S*_*mp*_, we performed 1,000 bootstraps (sampling with replacement) with the inserted-pollinium array as the unit of re-sampling. We used the mean and standard deviation of the 1,000 bootstraps as the mean and SE of *S*_*mp*_[8]. To compare the estimates of *S*_*mp*_ for the three visitor taxa, we used pairwise comparisons of the bootstrap estimates for each taxon and two-tailed tests. The *S*_*mp*_ for two visitor taxa were considered significantly different if > 975, or < 25, of the differences between randomly selected bootstrap estimates from the two visitor taxa were greater than zero [8,37,55].

We estimated pollinator effectiveness by subtracting the mean insertion rate (female function) in the control inflorescences from each of the visitor insertion values; we repeated the same step for removals (male function). If the resulting value was negative, it was set at zero [56]. Due to a large number of zeros, pollinator effectiveness was overdispersed, but we could not analyze pollinator effectiveness with a zero-inflated model because it is not count data. Therefore, we analyzed it using permutation tests, which have no assumptions about distribution or variance [57] and are appropriate for zero-inflated continuous data [58]. Permutation tests lack the standard parametric assumptions because they estimate p-values from distributions generated via Monte Carlo re-sampling of the data. We re-sampled our pollinator effectiveness data 1,000 times for each permutation test. In order to calculate the variance of pollinator importance and *SICPI*, we had to incorporate the variance of the product of two random variables for pollinator importance (pollinator effectiveness and pollinator relative abundance) and three random variables for *SICPI* (pollinator effectiveness, pollinator relative abundance and *S*_*mp*_). To estimate these variances we used a Monte Carlo simulation method in which we bootstrapped the relative abundance and pollinator effectiveness data 1,000 times and multiplied the mean of each abundance- and pollinator-effectiveness-bootstrap iteration to calculate pollinator importance [56]. We took the abundance- and pollinator-effectiveness-bootstrap means and combined them as described in eq. (4) for insertions and eq. (5) for removals with 1,000 bootstraps of the *S*_*mp*_ data to calculate *SICPI*. Then, for both pollinator importance and *SICPI* we calculated confidence intervals as the 25th and 975th values when the 1,000 values were ranked in ascending order. For all of the measures of pollinator effectiveness we controlled for sampling effort differences between diurnal and nocturnal visitors by adjusting visitation frequency according to the sampling effort ratio.

The negative-binomial and zero-inflated Poisson GLMs were log-linear models, and the quasi-binomial GLMM used a logit-link function. For all the parametric models, we analyzed the significance of the maximum likelihood-estimated or quasi-likelihood-estimated effects using likelihood ratio tests. We did not use any corrections for post hoc comparisons because there were at most three comparisons made between the three visitor taxa for each analysis resulting in a minimal increase in the probability of a type I error. We used R: A Language and Environment for Statistical Computing for all statistical analyses [59].

## Results

### Floral visitors

We observed a total of 408 individual insect visits, with 183 in 2008 and 225 in 2009. We observed visitation across the flowering season (early June through mid-July) during a total of 94 hr over 18 days in 2008, and 65 hr over 13 days in 2009. We observed hymenopterans (bees and kin), lepidopterans (butterflies and moths), dipterans (flies) and coleopterans (beetles) visiting *A. syriaca* flowers, finding that 94% of the 408 the visitors were hymenopterans and lepidopterans. The other visitors were not observed often enough (only 26 of the 408 visitors) for meaningful interpretation, so they were excluded from all analyses. We divided the remaining visitors into three taxonomic groups for the rest of the analyses: *Bombus* spp. (bumble bees), *A. mellifera* (Western Honey Bees), and lepidopterans (Table 1). *Bombus* spp. comprised 65%, *A. mellifera* comprised 18%, and lepidopterans comprised 10% of the 408 visitors. We observed *Bombus bimaculatus*, *Bombus griseocollis* (90% of all *Bombus* individuals), *B. impatiens*, and *B. vagans*. Diurnal lepidopteran visitors included skippers (Hesperiidae) and swallowtails (Papilionidae), and all of the nocturnal visitors were moths. The majority of the moths were crambids, geometrids, and noctuoids, but we also observed arctiids, and pyralids. Visitations by any family, genus, or species of lepidopterans were not frequent enough to enable analyses at lower taxonomic levels, and, at the qualitative level, the lepidopterans did not vary greatly between taxonomic family in visitation behavior, suggesting that the grouping by order was appropriate.

**Table 1:** **Identification of visitors included in the three visitor taxa: *Apis mellifera*, *Bombus *** **spp., and lepidopterans**^**1**^

**Order**	**Family**	**Genus**	**Species**	**Count**
Visitors collected				
Hymenoptera	Apidae	*Apis*	*mellifera*	51
		*Bombus*	*bimaculatus*	7
			*griseocollis*	139
			*impatiens*	2
			*vagans*	5
Lepidoptera	Arctiidae	*Lophocampa*	*caryae*	1
	Crambidae	*-*	*-*	6
	Geometridae	*-*	*-*	7
	Hesperiidae	*Pompeius*	*verna*	1
	Noctuidae	*Megalographa*	*biloba*	6
	Noctuidae	*-*	*-*	1
	Papilionidae	*Papilio*	*glaucus*	1
	Pyralidae	*-*	*-*	4
Visitors not collected				
Hymenoptera	Apidae	*Apis*	*mellifera*	24
		*Bombus*	spp.	118
Lepidoptera	-	*-*	*-*	12
	Hesperiidae	*Epargyreus*	*clarus*	2
	Papilionidae	*-*	*-*	1
		*Papilio*	*glaucus*	1

^1^Insect visitor’s quick movements at focal inflorescences often prevented us from collecting the visitors. We identified collected visitors to the lowest possible taxonomic level in our lab, and the rest to the lowest possible taxonomic level in the field.

### Visitor behavior and floral display

**Figure 1:**
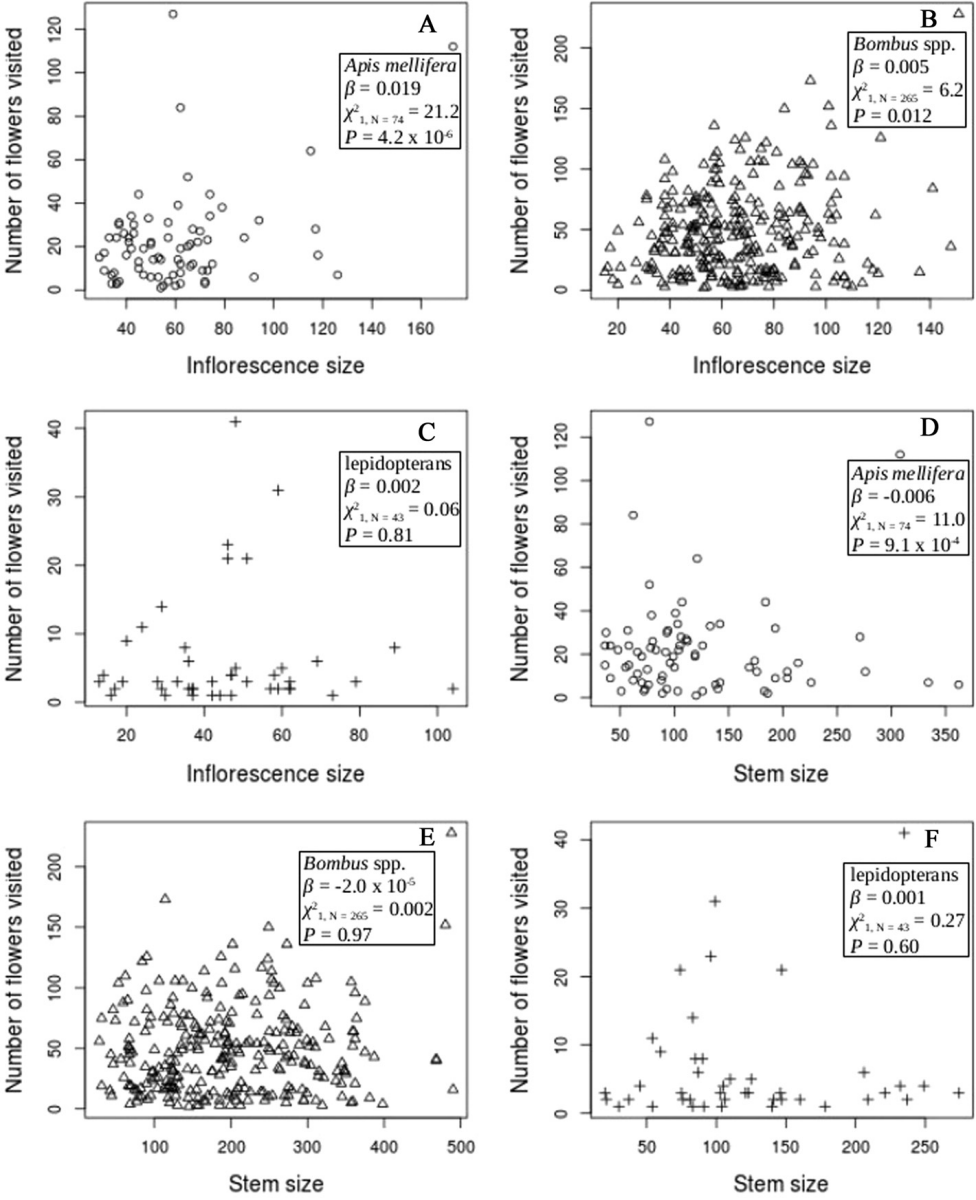
**Number of flowers visited versus inflorescence size and stem size for each visitor taxon. A** and **D**, *Apis mellifera* (circles); **B** and **E**, *Bombus* spp. (triangles); **C** and **F**, lepidopterans (crosses). Inflorescence size is the number of open flowers on the visited inflorescence, and stem size is the total number of open flowers on the visited stem. We estimated the coefficients (*β*) using a negative-binomial generalized log-linear model, and we calculated *χ*^2^s and *P*s using likelihood ratio tests to determine if *β*s were significantly different from zero.

There was a significant difference among visitor taxon in the number of flowers they visited ($\chi_{2}^{2}$_, N = 382_ = 27.4, *P* = 1.1 × 10^−6^, Figure 1). *Bombus* spp. visited the most flowers (48.5 ± 2.2), followed by *A. mellifera* (22.1 ± 2.5) and the lepidopterans (6.4 ± 1.3). In addition, there were interactive effects between visitor taxon and inflorescence size ($\chi_{2}^{2}$_, N = 382_ = 8.0, *P* = 0.02) and visitor taxon and stem size ($\chi_{2}^{2}$_, N = 382_ = 9.7, *P* = 0.01). The number of flowers visited increased at a greater rate as inflorescence size increased in *A. mellifera* than in *Bombus* spp. ($\chi_{1}^{2}$_, N = 339_ = 7.9, *P* = 0.005, Figure 1A and B), but not in lepidopterans. The number of flowers visited decreased as stem size increased, but at a greater rate in *A. mellifera* than *Bombus* spp. ($\chi_{1}^{2}$_, N = 339_ = 9.5, *P* = 0.002, Figure 1D and E). The ranges of stem size (*A. mellifera* = 36–362; *Bombus* spp. = 28–490; lepidopterans = 20–274), inflorescence size (*A. mellifera* = 29–173; *Bombus* spp. = 15–151; lepidopterans = 13–104), and the number of flowers visited (*A. mellifera* = 1–127; *Bombus* spp. = 1–228; lepidopterans = 1–41) overlapped for all three visitor taxa.

### Pollen deposition and removal

**Figure 2:**
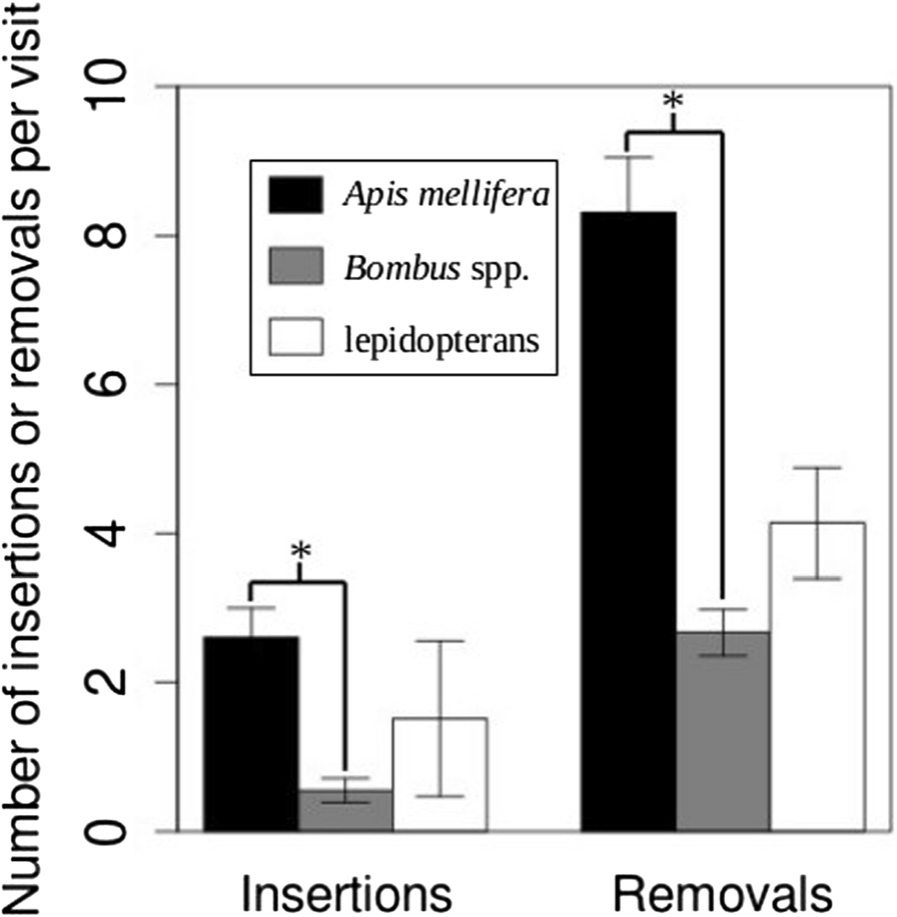
**The number of pollinium insertions and removals per visit (±1 SE) in *Apis mellifera *(black bars), *Bombus *spp. (gray bars), lepidopterans (open bars).** Asterisks indicate *P* < 0.05 for comparisons between visitor taxa in insertions or removals per visit. We calculated the *P* values using likelihood ratio tests that compared coefficients (*β*) estimated from a zero-inflated Poisson generalized log-linear model.

**Figure 3:**
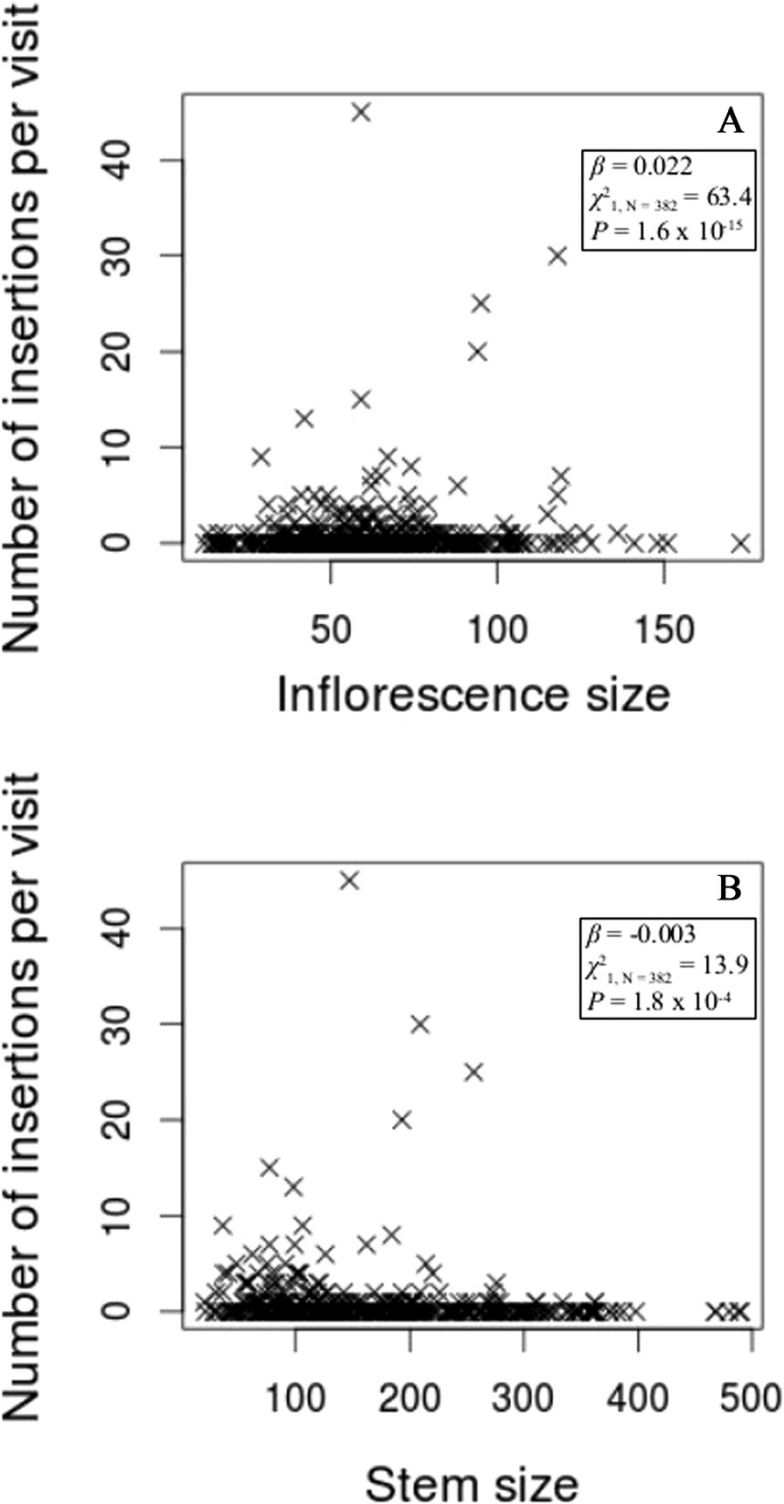
**The number of pollinium insertions per visit versus inflorescence size (A) and stem size (B).** Inflorescence size is the number of open flowers on the visited inflorescence, and stem size is the total number of open flowers on the visited stem. We estimated the coefficients (*β*) using a zero-inflated Poisson-generalized log-linear model, and we calculated *χ*^2^s and *P*s using likelihood ratio tests to determine if *β*s were significantly different from zero.

The number of pollinia inserted per visit in *A. mellifera*, but not *Bombus* spp. and lepidopterans, was significantly greater than the insertions per visit in the control inflorescences ($\chi_{1}^{2}$_, N = 125_ = 5.2, *P* = 0.023). There was a significant effect of visitor taxon on insertion rate ($\chi_{2}^{2}$_, N = 382_ = 16.9, *P* = 2.1 × 10^−4^). *Apis mellifera* had the greatest insertion rate per visit (2.61 ± 0.39), followed by lepidopterans (1.51 ± 1.05) and *Bombus* spp. (0.55 ± 0.16) (Figure 2, Additional file 1: Table S1). Overall, there was a significant influence of inflorescence size ($\chi_{1}^{2}$_, N = 382_ = 63.4, *P* = 1.6 × 10^−15^, Figure 3A) and stem size ($\chi_{1}^{2}$_, N = 382_ = 13.9, *P* = 1.8 × 10^−4^, Figure 3B). The number of insertions per visit increased as inflorescence size increased, and the number of insertions per visit decreased as stem size increased. There was no significant relationship between the number of insertions in the control inflorescences and floral-display size. For the binomial component of the zero-inflated model, visitor taxon was a statistically significant predictor of the excess number of visits (relative to the Poisson-distribution expectation) that resulted in zero insertions ($\chi_{1}^{2}$_, N = 382_ = 49.2, *P* = 2.0 × 10^−11^).

**Figure 4:**
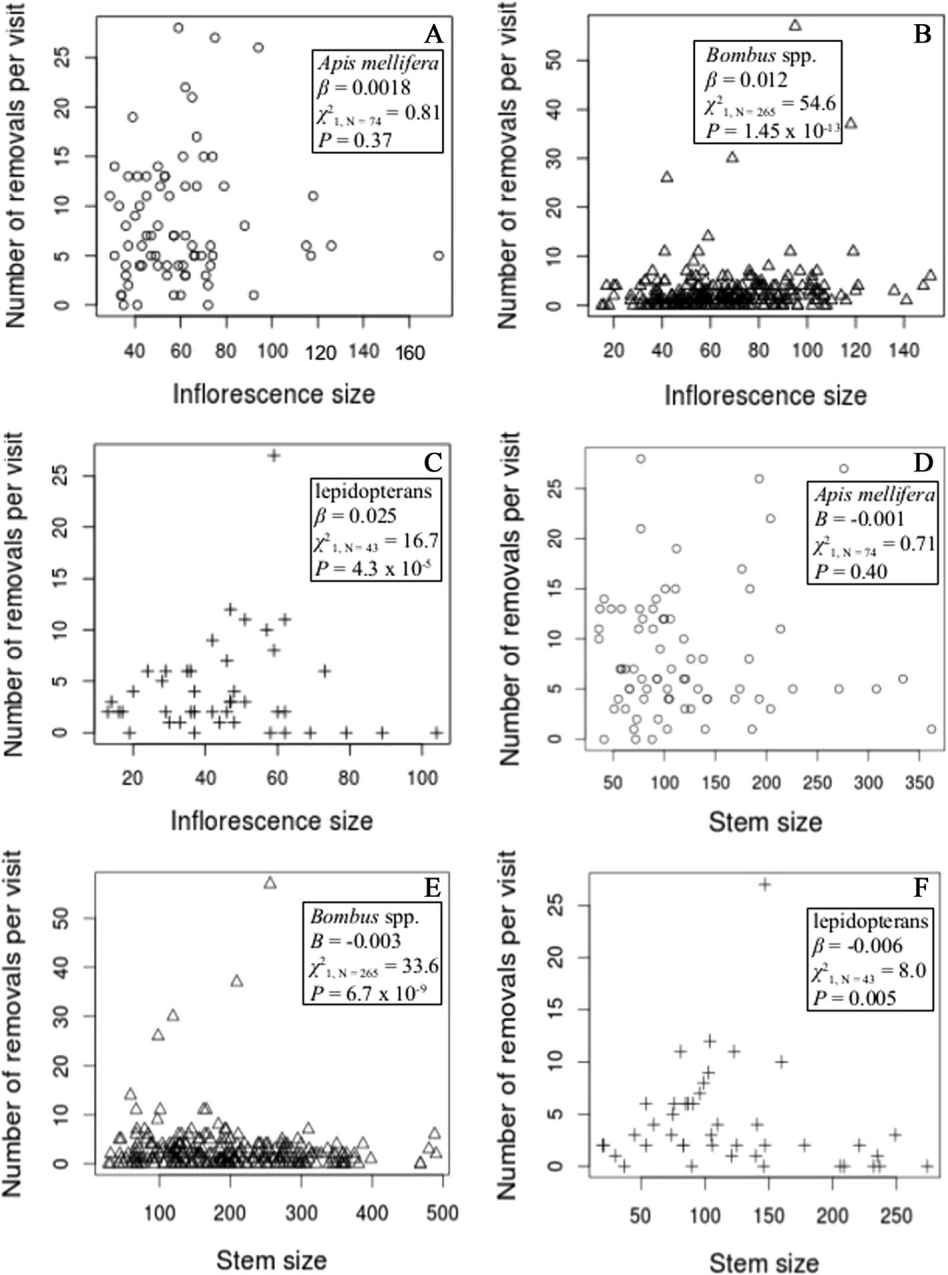
**The number of pollinium removals per visit versus inflorescence size and stem size for each visitor taxon. A** and **D**, *Apis mellifera* (circles); **B** and **E**, *Bombus* spp. (triangles); **C** and **F**, lepidopterans (crosses). Inflorescence size is the number of open flowers on the visited inflorescence and stem size is the total number of open flowers on the visited stem. We estimated the coefficients (*β*) using a zero-inflated Poisson-generalized log-linear model, and we calculated *χ*^2^s and *P*s using likelihood ratio tests to determine if *β*s were significantly different from zero.

The number of pollinia removed per visit by *A. mellifera* and *Bombus* spp., but not lepidopterans, was significantly greater than the removal rate in the control inflorescences ($\chi_{1}^{2}$_, N = 125_ = 75.3, *P* < 2.2 × 10^−16^ and $\chi_{1}^{2}$_, N = 308_ = 10.8, *P* = 9.7 The number of pollinia removed 10^−4^, respectively). Visitor taxon had a significant effect on removals per visit ($\chi_{2}^{2}$_, N = 382_ = 189.9, *P* < 2.2. The number of pollinia removed 10^−16^). *Apis mellifera* had the greatest removal rate per visit value (8.32 ± 0.74), followed by lepidopterans (4.14 ± 0.75) and *Bombus* spp. (2.67 ± 0.31) (Figure 2, Additional file 1: Table S1). There was a significant interaction between visitor taxon and inflorescence size ($\chi_{1}^{2}$_, N = 382_ = 13.4, *P* = 8.1 The number of pollinia removed 10^−6^, Figure 4A, B, and C). Removals per visit increased with inflorescence size in all taxa but at the greatest rate for lepidopterans, followed by *Bombus* spp. and *A. mellifera*. Also, there was a significant interaction between visitor taxon and inflorescence size ($\chi_{1}^{2}$_, N = 382_ = 8.1, *P* = 0.017, Figure 4D, E, and F). Removals per visit decreased with stem size in all taxa but at the greatest rate for lepidopterans, followed by *Bombus* spp. and *A. mellifera*. There was no significant relationship between the number of removals from the control inflorescences and floral-display size. For the binomial component of the zero-inflated model, visitor taxon was a statistically significant predictor of the excess number of visits (relative to the Poisson distribution expectation) that resulted in zero removals ($\chi_{1}^{2}$_, N = 382_ = 14.7, *P* = 6.2 × 10^−4^).

### Self-pollination rate

We successfully genotyped a total of 91 inserted pollinia, and there were a total of 22 unique maternal genotypes and 21 pollinium genotypes. The average most common genotype per locus for maternal plants and pollinia were 0.49 (SE ± 0.05) and 0.64 (SE ± 0.06), respectively (Additional file 2: Table S2). Mean α was 0.28 (SE ± 0.07), and *Bombus* spp. had the lowest value (0.05 ± 0.12, Additional file 2: Table S2). While much of the difference between taxa in α was due to sample size differences, there was still a difference when controlling for sample size (α in *A. mellifera* = 0.31; *Bombus* spp. = 0.17; lepidopterans = 0.72). There was a small change in α as a result of the round-robin method of allele frequency estimation (Additional file 2: Table S2).

**Figure 5:**
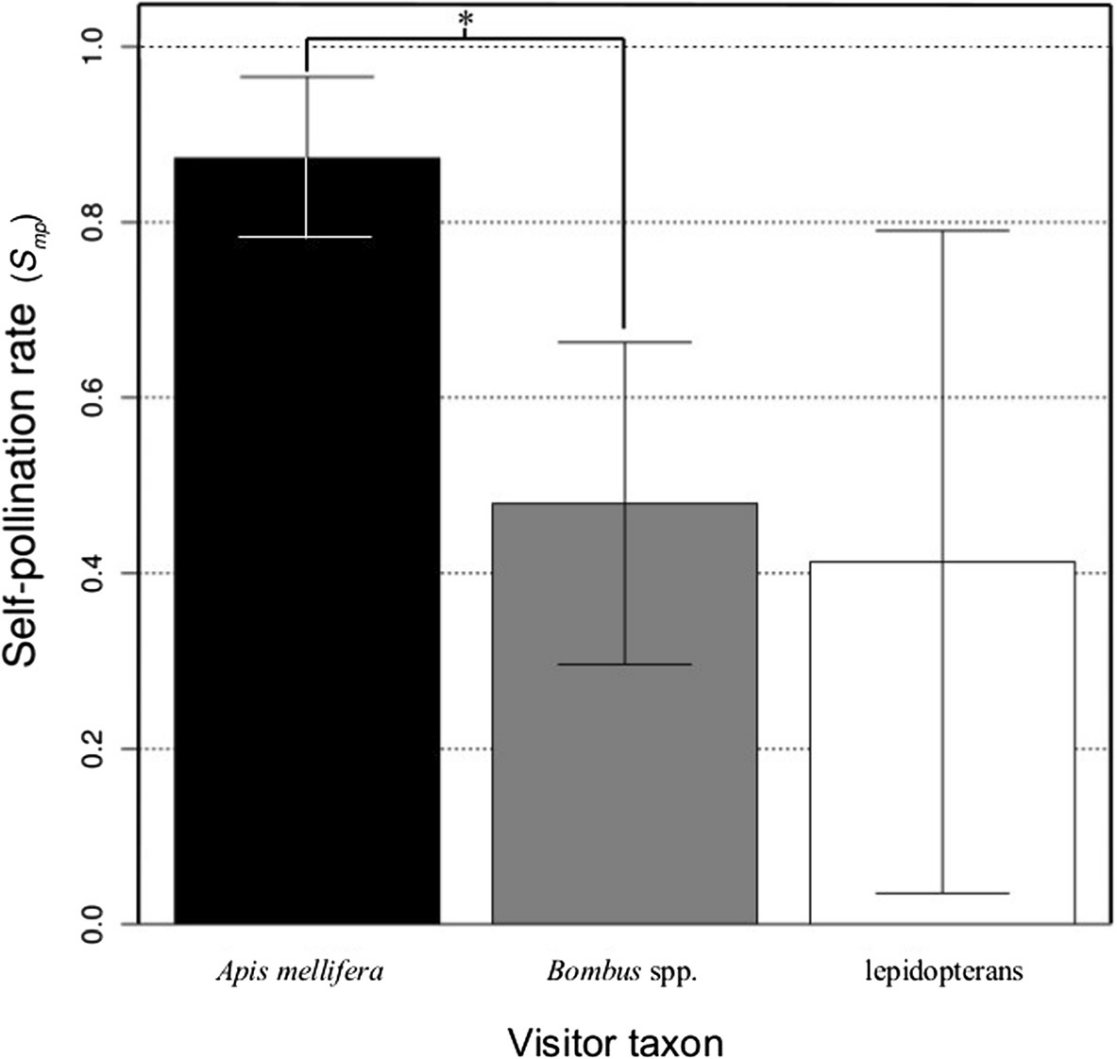
**Modified method-of-moments self-pollination rate (*S***_***mp ***_**± 1 SE) in *Apis mellifera*, *Bombus *spp., and lepidopterans.** All maternal genotypes (*i.e.*, inflorescences with the same genotype) visited by a taxon were used to estimate its influence on *S*_*mp*_. *Apis mellifera* visited 13 maternal genotypes, *Bombus* spp. visited 16, and lepidopterans visited four. The asterisk indicates *P* < 0.05 for comparisons between visitor taxa in insertions or removals per visit. We calculated the *P*s from pairwise comparisons of the bootstrap estimates for each taxon. We considered the *S*_*mp*_ for two visitor taxa to be significantly different if > 975, or < 25, of the differences between randomly selected bootstrap estimates from the two visitor taxa were greater than zero.

For the method-of-moments estimation of self-pollination (*S*_*mp*_), *A. mellifera* had the highest self-pollination rate (0.88 ± 0.1), followed by *Bombus* spp. (0.49 ± 0.19), and the lepidopterans (0.41 ± 0.38) (Figure 5). The bootstrap estimated mean of *S*_*mp*_ in *A. mellifera* was significantly greater than the bootstrap estimated mean of *S*_*mp*_ in *Bombus* spp. The rest of the comparisons (*A. mellifera* and lepidopterans; *Bombus* spp. and lepidopterans) were not statistically different.

**Figure 6:**
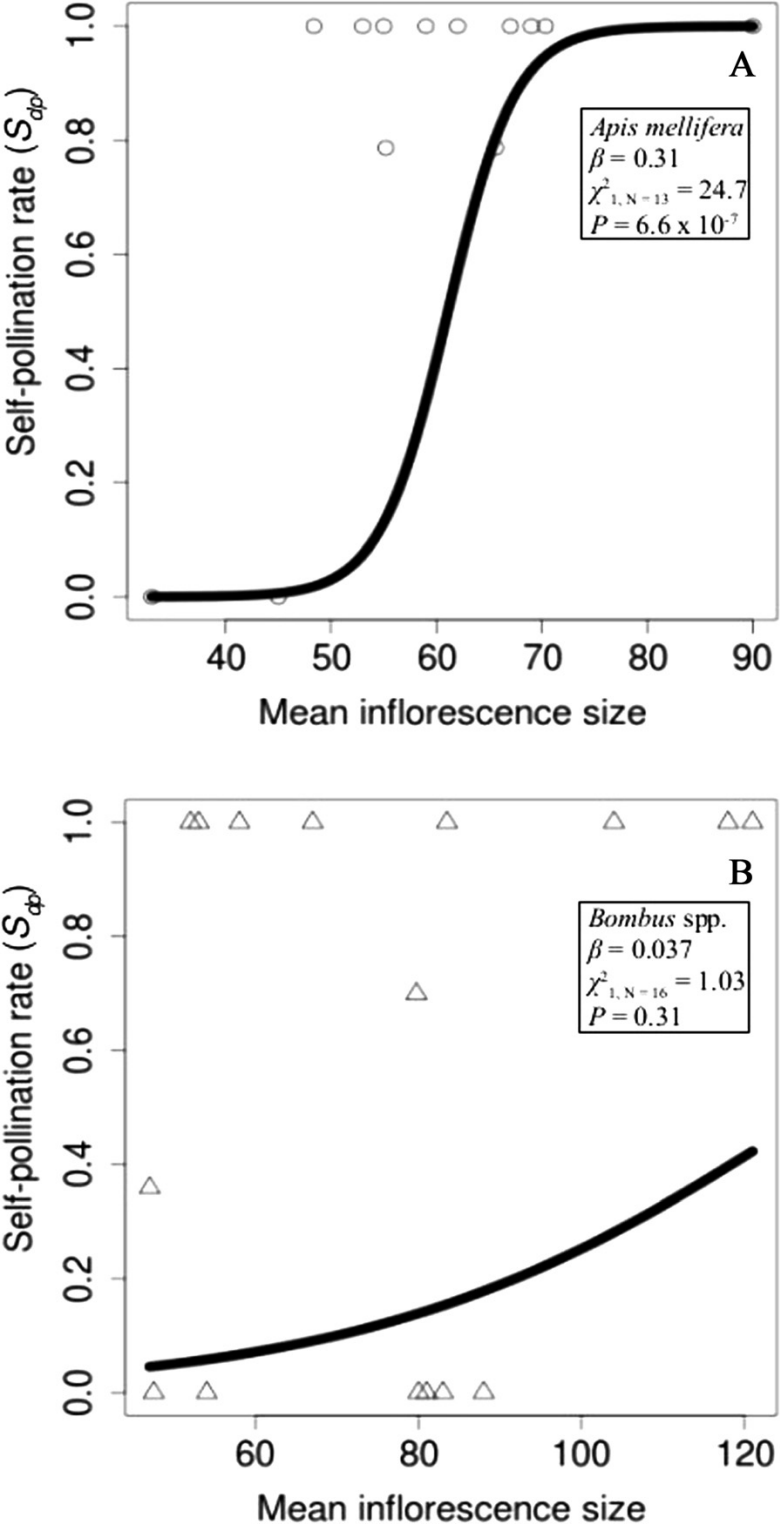
**Direct self-pollination rate (*S***_***dp***_**) versus mean inflorescence size in *Apis mellifera *(A) and *Bombus *spp. (B).** We estimated *S*_*dp*_ for each maternal genotype (*i.e.*, inflorescences with the same genotype), and calculated mean inflorescence size as the average number of open flowers on all inflorescences with the same maternal genotype. For both plots, we based the best-fit lines on coefficients (*β*) estimated using a quasi-binomial mixed model (GLMM) with a logit-link function. We calculated *χ*^2^s and *P*s using likelihood ratio tests to determine if the *β*s were significantly different from zero. Many of the *S*_*dp*_ values were 0 or 1 because of one or any combination of the following reasons: most inflorescences had a single pollinium inserted (the mode value for insertion rate was one pollinium), the multiple pollinia inserted into a single inflorescence were either all self- or all outcrossed pollinia, and we successfully genotype only one of the pollinia inserted into an inflorescence due to the extremely small amount of genetic material in each pollinium. The mean inflorescence sizes in *A. mellifera* and *Bombus* spp. were markedly different, but the range of inflorescence sizes (*A. mellifera* = 29–173; *Bombus* spp. = 15–151) overlapped.

Due to a limited number of *A. syriaca* maternal genotypes (a total of 4) visited by lepidopterans and extreme variability in their insertion rates, lepidopterans were omitted from the analysis of *S*_*dp*_ and floral-display size. There was an overall significant difference in the direct self-pollination rate (*S*_*dp*_) between *A. mellifera* and *Bombus* spp. ($\chi_{1}^{2}$_, N = 21_ = 18, *P* = 2.1 × 10^−5^, Additional file 1: Table S1). Also, there were several statistically significant relationships between *S*_*dp*_ and floral-display size including a significant overall effect of stem size on *S*_*dp*_ ($\chi_{1}^{2}$_, N = 21_ = 5.5, *P* = 0.019), and an interaction between visitor taxon and inflorescence size ($\chi_{1}^{2}$_, N = 21_ = 21.6, *P* = 3.4 × 10^−6^). Specifically, *S*_*dp*_ increased as stem size increased and increased as inflorescence size increased at a greater rate in *A. mellifera* than in *Bombus* spp. (Figure 6A and B).

### Pollinator effectiveness and importance

**Figure 7:**
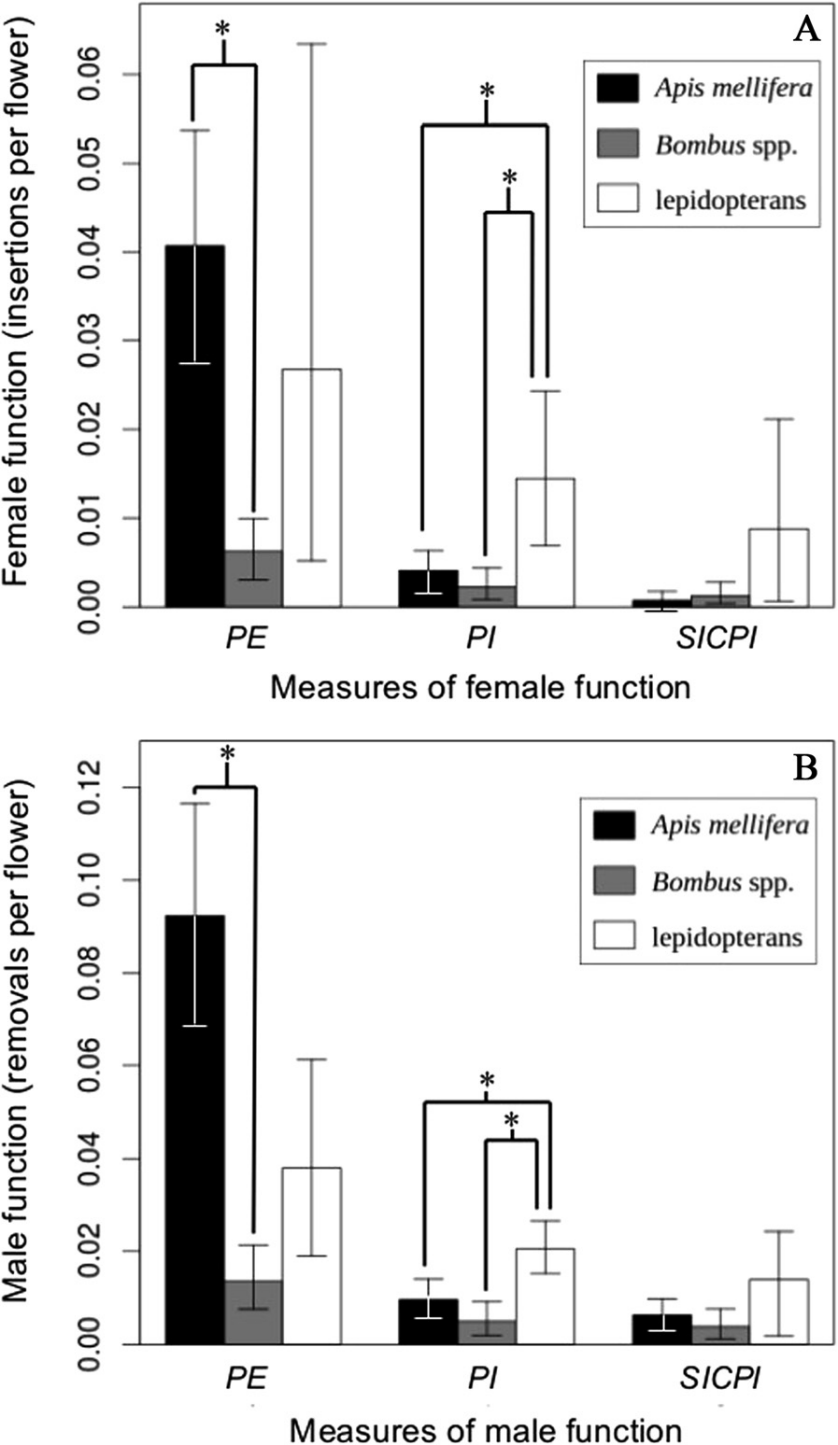
***Asclepias syriaca *female function and male function pollinator effectiveness (*PE*), pollinator importance (*PI *), and self-incompatibility-controlled pollinator importance (*SICPI*) versus visitor taxon. A**, measures of pollinator effectiveness for female function (calculated using pollinium insertions); **B**, measures of pollinator effectiveness for male function (calculated using pollinium removals). Black bars, *Apis mellifera*; gray bars, *Bombus* spp.; open bars, lepidopterans. The unit for *PI* and *SICPI* are the same as *PE*, insertions per flower on the focal inflorescence. The error bars for *PE*, *PI*, and *SICPI* are 95% confidence intervals (C.I.). Asterisks indicate *P* < 0.05 for comparisons between visitor taxa in *PE*, *PI*, or *SICPI*. Comparisons for *PE* were based on permutation tests generated from Monte Carlo re-sampling. Comparisons for *PI* and *SICPI* were based on mean and C.I. estimates. The mean male and female pollinator importance (*PI*) values in *A. mellifera* and *Bombus* spp. were not within the 95% C.I. of lepidopterans; therefore, pollinator importance in lepidopterans was significantly greater than pollinator importance in *Bombus* spp. or *A. mellifera*. This result is inconsistent with our pollinium-insertion data and may be due to the fact that lepidopterans had the smallest average inflorescence size (pollinator effectiveness is calculated as insertions or removals per focal-inflorescence flower) and that we controlled for sampling effort differences between diurnal and nocturnal visitors when calculating relative abundance (pollinator importance is pollinator effectiveness multiplied by relative abundance). In other words, we collected fewer nocturnal samples, and all nocturnal visitors were lepidopterans.

There was a significant overall effect of visitor taxon on pollinator effectiveness through female (*t*_N = 382_ = 4.9, *P* = 1.0 × 10^−4^) and male (*t*_N = 382_ = 7.5, *P* < 2.2 × 10^−16^) reproduction, measured using insertions and removals, respectively. Pollinator effectiveness was significantly greater in *A. mellifera* than *Bombus* spp. through both female (*z*_N = 339_ = 6.6, *P* < 2.2 × 10^−16^, Figure 7A) and male (*z*_N = 339_ = 7.6, *P* < 2.2 × 10^−16^, Figure 7B) reproduction. The mean male and female pollinator importance values in *Bombus* spp. and *A. mellifera* were not within the 95% C.I. in lepidopterans; therefore, pollinator importance in lepidopterans was significantly greater than pollinator importance in *Bombus* spp. or *A. mellifera*. Male and female self-incompatibility-controlled pollinator importance (*SICPI*) was not statistically different between any of the visitor-taxon pairs for male or female reproduction. However qualitatively, lepidopterans and *Bombus* spp. had greater importance for female reproduction than *A. mellifera* when considering visitor abundance, self-pollination, and self-incompatibility (Figure 7A). For the diurnal visitors, *Bombus* spp. was the most important pollinator through female reproduction, and *A. mellifera* was the most important through male reproduction.

## Discussion

Our study quantified floral-visitor behavior, pollen deposition, and the self-pollination rates of *A. syriaca* for the three visitor taxa, *A. mellifera*, *Bombus* spp., and lepidopterans*.* Our results are in agreement with previous studies that the majority of diurnal pollinators are hymenopterans [7,17,18,24-26] and that *A. syriaca* is generalist pollinated [7,18]. In addition, we found differences in the behavior, pollen movement, and the self-pollen deposition by visitors, which correlated with floral-display size.

Lepidopteran-visited inflorescences had the greatest variability in pollen deposition and removal through male and female function (Figures 2 and 7), resulting in no statistical difference between the insertion and removal rates for such inflorescences and the control inflorescences. While this large variability may be explained by the lepidopterans’ being our most taxonomically diverse group, we could not separate it into subgroups because of the relative infrequency of each family’s or species’ visitations. In addition, lepidopterans did not have more variance in their visitation behavior than the other visitor taxa, except in visiting time. Lepidopterans spend the most time per visit on the focal inflorescence which may be due to increased nectar volume and sucrose production in *A. syriaca* at night [26,60,61]. We observed only one lepidopteran visitor, a *Lophocampa caryae* (Hickory Tussock Moth), which had a pollinium attached to its body, in our study areas. Adults of this species are large, nocturnal visitors with the size and strength to remove and potentially insert pollinia; but many of the nocturnal lepidopteran visitors were small moths (*e.g*., crambids, geometrids, and pyralids) that may not be capable of removing pollinia. However, we included the small moths in the lepidopteran visitor group because there is evidence that small nocturnal moths insert and remove pollinia in some asclepiadoid species [62]. Future studies should examine different lepidopteran floral visitors to understand their roles in *A. syriaca* pollination.

Regarding self-pollination, there was no statistical difference between lepidopteran *S*_*mp*_ and *A. mellifera* or *Bombus* spp. *S*_*mp*_ (Figure 5), but qualitatively it was between *A. mellifera* and *Bombus* spp. *S*_*mp*_. Lepidopterans were the least important diurnal visitors, which is consistent with past studies of diurnal *Asclepias* pollinators [7,18]. Bertin and Willson [63] showed that nocturnal pollinators deposited less pollen, but produced the same number of fruit as diurnal pollinators and suggested that nocturnal pollinators carried higher-quality pollen than diurnal pollinators. Morse and Fritz [60] found that nocturnal pollinators were less likely to deposit pollinia or produce surviving fruit. Our results are comparable with the results of Bertin’s and Willson’s study [63], which determined nocturnal pollinators are important, but inconsistent with their studies’ pollinium-insertion results. This suggests that there may be great variability among populations in pollinator-mediated pollen deposition.

*Apis mellifera* had the highest rate of pollen deposition and removal per visit and the highest pollinator effectiveness (Figure 7). *Bombus* spp. had the fewest insertions and removals per visit and was the least effective pollinator (Figure 7) despite the fact that they visited stems and inflorescences with the greatest number of flowers. These results demonstrate that the important difference between the influence of *A. mellifera* and *Bombus* spp. on plant reproduction is most likely in the efficiency of pollen deposition. This conclusion is also supported by the interesting relationship between floral-display size and the rate of self-pollination. *Apis mellifera* had the highest self-pollination rate (Figure 5), which increased with inflorescence size at a greater rate than in *Bombus* spp. (Figure 6). The increase in self-pollination with inflorescence size suggests that, due to its efficiency of pollen movement (in terms of insertions and removals), *A. mellifera* exhaust outcrossed pollen that they carryover from other plants very quickly and, consequently, tend to remove pollinia from and deposit them on the same inflorescence.

While there is a significant difference between *A. mellifera* and *Bombus* spp. in the number of pollinia they insert and remove, there are some inconsistent differences in the rate of change in insertions and removals as floral-display size increases; for example, insertions per visit increased as inflorescence size increased for both taxa at the same rate. This, again, may be due to differences in the efficiency of pollen deposition and removal. Both *A. mellifera* and *Bombus* spp. removed pollinia at a greater rate than they inserted them, but the disparity is more significant in *A. mellifera* (Figure 2)*,* resulting in a greater accumulation of pollinia on *A. mellifera* individuals. Carrying pollinia has been shown to decrease the foraging speed of bumble bees, increase difficulties in foraging (*e.g*., losing footing and freeing body parts from flowers), and cause movement to new stems more frequently [64]. So at the beginning of each visit to an inflorescence, *A. mellifera* may be inserting and removing pollinia faster than *Bombus* spp., which accounts for the significantly higher insertion and removal rates in *A. mellifera*; but, as the number of flowers visited increases *A. mellifera* may become less efficient in its pollen movement than *Bombus* spp. The increased pollinium load on *A. mellifera* individuals may eliminate any potential mean differences between the two taxa in the dynamics of pollen deposition and removal due to floral-display size. This conclusion is supported by the observations that *Bombus* spp. visited more flowers than *A. mellifera* (Figure 1), as the foraging time increased the number of flowers visited leveled off in *A. mellifera* (pers. obs.), *A. mellifera* had a smaller increase in removals with inflorescence size as compared to the other visitor taxa (Figure 4), *A. mellifera* had the smallest number of predicted excess zeros (relative to the Poisson-distribution expectation) in the insertion and removal zero-inflated models, and in other populations, as in our population, *A. mellifera* had more pollinia attached to their bodies than any other visiting species [65,66].

Stem size also had a significant influence on visitor behavior (Figure 1), pollen deposition and removal, and the self-pollination rates. The number of insertions and removals decreased with stem size (Figures 3 and 4), but the self-pollination rate increased. These results are consistent with previous research that showed an influence of nearby flower density on visiting behavior [1,32,67], but the relationships between these variables and stem size are weaker than their relationships with inflorescence size. For example, the overall effect of inflorescence size (*β* = 0.31) on the self-pollination rate is larger than the overall effect of stem size (*β* = 0.02), which is a significant difference despite the fact that these are coefficients in a model with a logit-link function.

Another possible explanation for the disparity between the self-pollination rates of *A. mellifera* and *Bombus* spp. could be different movement patterns among stems. Our results for stem size suggest that larger stem floral-display size may reduce the number of insertions within an inflorescence and, if stem size is a proxy for patch size, promotes movement among stems within a patch. This would mean that the pollen deposited by *A. mellifera* could represent a high self-pollination rate from other stems of the same clone within a patch. *Apis mellifera* preferentially visit patches with large neighborhood sizes [68] and frequently move between nearest neighbors regardless of interplant distance [69] suggesting that the spatial aggregation of ramets within a clone (*i.e*.,clonal spatial autocorrelation) of *A. syriaca* could result in extremely high self-pollen carryover. Nonetheless, these results are comparable with studies that have demonstrated that bumble bees preferentially visit inflorescences with higher inflorescence size (*e.g*., ref [1]) and patches with higher floral-display density [67]. Despite the apparent similarities in their movement among stems [32,67,68], it is important that future studies consider pollinator movement among stems in order to examine its possible effects on self-pollination rates. This is especially important for populations, like ours, with relatively large clone sizes, and our genetic data indicate large clones due to the relatively few unique genotypes found and the small difference between the observed and round-robin estimates of α (probability of erroneous pollen source assignment).

*Apis mellifera*’s high α value as compared to that in *Bombus* spp. (Additional file 2: Table S2) seems to be consistent with the differences found in their self-pollination rates (*S*_*mp*_) (Figure 5). While an argument can be made for using a single α value for estimating the self-pollination rate of all three visitor taxa, separate estimates give more realistic measures of the pollen-pool allele frequencies for each pollinator and a conservative estimate of the difference between the two taxa because a single α value for both taxa would have resulted in a higher estimate of *S*_*mp*_ in *A. mellifera* and a lower estimate in *Bombus* spp. Additionally, an argument can be made that using more than four microsatellite loci would lower α and improve the self-pollination rate estimates. However, our *Bombus* spp. α value suggests that our power of exclusion is adequate, and increasing the number of loci genotyped was logistically impossible due to the small size of the pollinia. The higher α value in lepidopterans is indicative of the extremely variable rates of pollen deposition and the concomitant limited number of pollinium genotypes for this plant.

*Apis mellifera*’s significantly higher self-pollination rate (*S*_*mp*_) as compared to that in *Bombus* spp. (Figure 5) suggests that comparing simple measures of pollinator effectiveness alone is a biased method for determining the differential influence of these pollinators on plant reproduction. *Apis mellifera* is still a 30% more effective pollinator than *Bombus* spp., considering the actual quantity of self- versus outcross pollen deposited per pollinator per flower. However, when pollinator abundance, self-pollination rate, and self-incompatibility (*SI*) are included there is no difference between these bees' pollinator importance values (Figure 7). Additionally, the relative-abundance-controlled number of self-pollinium depositions per flower was three times greater in *A. mellifera* than *Bombus* spp. Therefore, despite the greater abundance of *Bombus* spp. pollinators, *A. mellifera* were still responsible for a larger portion of the self-pollinium insertions. This pattern is consistent with what we found for pollinium removals (Figure 7) meaning that the consequences of self-pollination may be similar for both male and female reproduction.

The high rate of self-pollination along with the derived floral morphology, which prevents non-vector autogamy in *A. syriaca*, means that a very high percentage of pollination events in our study were geitonogamous insertions made by insects. Geitonogamy is viewed as disadvantageous when plants are self-incompatible because it results in pollen discounting [9,70] and reduced seed set [71]. *Asclepias syriaca* is highly self-incompatible, and our measure of pollinator importance through male reproduction that controls for self-incompatibility (*SICPI*_*r*_) suggests that pollen discounting could be very high in our study populations, for example, SICPI_*r*_ in *A. mellifera* is ~37% less than male function pollinator importance (Figure 7B). Self-incompatibility is a mechanism which prevents reduction in fruit production due to self-pollination, indicating that if our populations were not pollen limited the negative consequences of self-pollination for female reproduction may not be significant [9]. On the other hand, the limited number of stigmatic slits of *A. syriaca* flowers may increase the probability of reduced fruit production despite the quantity of pollen available [24].

If *A. syriaca* populations were pollen limited, geitonogamous pollinations may be an inevitable consequence of selection for increased floral-display size and pollinator visitation. But even if an *A. syriaca* population is pollen limited, there may still be a point where the detrimental effects on male and female fertility outweigh the benefits of increased floral-display size [13,72]. Pollen limitation in *A. syriaca* is still under investigation, but in our populations the insertion rate for open pollinated inflorescences was high (1.5 insertions per flower). This high insertion rate, along with our population estimate of the self-pollination rate of 0.54 and the mean inflorescence size of 62.5, results in an estimate of over 42 compatible inserted pollinia per inflorescence, which is much larger than the mean number of observed fruit per inflorescence (0.93, N = 76, pers. obs.), suggesting that there was no pollen limitation. Unfortunately, pollen limitation is methodologically difficult to estimate [73,74] and probably varies spatially and temporally within a species [24].

The rapid rate of self-pollen deposition by *A. mellifera* may be due to the limited time for co-evolution between this bee and *A. syriaca* in North America, where Colonists introduced *A. mellifera* about 400 yr ago [75], whereas *Bombus* spp. has likely interacted with *A. syriaca* for thousands of years. However, we found that *A. mellifera* visits *A. syriaca* stems less frequently than *Bombus* spp. (Table 1), which might lessen possible selection on floral-display size by the former compared to the latter, at least in our study areas. To our knowledge, there are no studies of how *A. mellifera* might have changed *A. syriaca* floral-display size in North America or in Europe, where this plant has naturalized over the centuries.

Whether or not there is a single target of selection on floral-display size, be it the inflorescence, total plant, or some combination of multiple measures of floral-display size is still unknown in *Asclepias*[21]. Some evidence supports a relationship between all levels of floral-display size and reproductive success [19,23], suggesting that there may be multiple targets of selection. In our study, pollinator behavior, pollinium insertions and removals, and the self-pollination rate did vary with inflorescence size and stem size (Figures 1, 3, 4, and 6), but it was not possible for us to determine how clone (whole-plant) floral display influences pollen deposition because clones interdigitate and we did not identify every stem of every clone within our study populations. Because stems of clones are generally highly interdigitated with those of other clones, pollinators may not be able to visually differentiate between stems of different plants [76] and, therefore, clone-floral-display size may not influence pollinator behavior as strongly as other levels of floral-display size, such as stem and inflorescence displays.

Beyond visual discrimination between plants based on floral-display size, pollinators can differentiate between plants based on floral characteristics such as nectar quality and quantity and flower size and shape [77]. This ability to discriminate is important because these characteristics may help explain our visitation-behavior and pollinium-deposition results. There is a paucity of information on the influence of floral characters, other than display size, on the behavior of *Asclepias* visitors, but there is evidence that nectar characteristics can affect pollination and fruit production [78]. For example, nectar volumes per flower decrease in *Asclepias quadrifolia* as inflorescence size increases [79] and may be a result of limited resources or an evolutionary strategy that maximizes pollen export by reducing the proportion of flowers visited and the deposition of geitonogamous pollen [80,81]. A negative relationship between nectar production and inflorescence size in *A. syriaca* could explain why the number of flowers visited does not increase proportionately with inflorescence size (Figure 1) in our populations and mitigate the frequency and detrimental effects of geitonogamous-pollinium depositions.

## Conclusions

In summary, we directly quantified the self- and outcross origins of deposited pollen in *A. syriaca* and examined the relationships between floral-visitor behavior, self-pollination, and floral-display size. We found significant differences among visitor taxa in behavior, pollen deposition, and self-pollination rates. The difference in self-pollination rate between *A. mellifera* and *Bombus* spp. occurred with regards to inflorescence size, suggesting differential selection pressures applied by the two taxa on this character. Additionally, our results agree with those of Goulson’s study [27], that *A. mellifera* is very efficient at depositing pollen, but our results show that *A. mellifera* deposited the largest proportion of self-pollen of our three study taxa. This phenomenon and the fact that the introduced *A. mellifera*[75] does not facilitate plant reproduction as well as native pollinators [82], suggest there is a disconnect between the quantity and quality of pollen deposited by *A. mellifera,* which could strongly influence the reproductive success and evolutionary trajectory of *A. syriaca*.

## Abbreviations

α: Probability of erroneous pollen source assignment; *PE*: Pollinator effectiveness; *PI*: Pollinator importance; *S*: Self-pollination rate; *SICPI*: Self-incompatibility-controlled pollinator importance.

## Competing interests

The authors declare that they have no competing interests.

## Authors’ contributions

AFH conceived the study, performed the field work, microsatellite analysis, and statistical analyses and was the lead writer. EMB participated in the study design and coordination and was the second writer. Both authors read and approved the final manuscript.

## Supplementary Material

Additional file 1: Table S1 Raw data for each inflorescence visited by *Apis mellifera* or *Bombus* spp. Sample number, visitor taxon, number of flowers visited, number of flowers on the inflorescence (inflorescence size), number of flowers on the inflorescence’s stem (stem size), number of pollinium insertions (number of insertions), number of pollinium removals (number of removals), and the direct self-pollination rate^1^ (*S*_*d*_) are included for each inflorescence that was visited by *A. mellifera* or *Bombus* spp. and had at least one pollinium genotyped. Click here for file

Additional file 2: Table S2 Means (± SE) of the maternal (M) and inserted-pollinium (I) genotypic data for the three visitor taxa across four polymorphic-microsatellite-locus primer sequences. α is the probability of erroneous pollen-source assignment. Round-robin values were calculated using the methodology described in Park and Werth [51]. Alleles per locus, frequency of the most common allele per locus, genotypes per locus, and frequency of the most common genotype per locus are averaged across four polymorphic-microsatellite-locus primer sequences from O’Quinn and Fishbein [48] (Asyr-C4, Asyr-C102, Asyr-C103, Asyr-C109). The overall values are sample-size-weighted means of the values calculated for each visitor taxon. Click here for file
