# Supplementary material for: Self-pollination rate and floral-display size in Asclepias syriaca (Common Milkweed) with regard to floral-visitor taxa
Source: BMC Evol Biol. 2014 Jun 23;14:144. doi: 10.1186/1471-2148-14-144 (PMC4080991; doi:10.1186/1471-2148-14-144)
Supplement: Additional file 1: Table S1 — Raw data for each inflorescence visited by Apis mellifera or Bombus spp. Sample number, visitor taxon, number of flowers visited, number of flowers on the inflorescence (inflorescence size), number of flowers on the inflorescence’s stem (stem size), number of pollinium insertions (number of insertions), number of pollinium removals (number of removals), and the direct self-pollination rate1 (Sd) are included for each inflorescence that was visited by A. mellifera or Bombus spp. and had at least one pollinium genotyped. [file 1471-2148-14-144-S1.doc]

**Additional file 1: Table S1. Raw data for each inflorescence visited by *Apis mellifera* or *Bombus* spp.** Sample number, visitor taxon, number of flowers visited, number of flowers on the inflorescence (inflorescence size), number of flowers on the inflorescence's stem (stem size), number of pollinium insertions (number of insertions), number of pollinium removals (number of removals), and the direct self-pollination rate^1^ (*S_d_*) are included for each inflorescence that was visited by *A. mellifera* or *Bombus* spp. and had at least one pollinium genotyped.

| Sample  number | Visitor taxon | Number of flowers visited | Inflorescence size | Stem size | Number of insertions | Number of removals | *S_d_* |
| --- | --- | --- | --- | --- | --- | --- | --- |
| 2 | *Apis mellifera* | 38 | 79 | 79 | 4 | 12 | 1 |
| 4 | *Apis mellifera* | 24 | 57 | 58 | 3 | 7 | 1 |
| 5 | *Apis mellifera* | 28 | 67 | 106 | 9 | 12 | 1 |
| 9 | *Apis mellifera* | 24 | 88 | 126 | 6 | 8 | 1 |
| 17 | *Bombus* spp. | 58 | 67 | 67 | 4 | 6 | 1 |
| 18 | *Apis mellifera* | 11 | 66 | 66 | 1 | 5 | 1 |
| 25 | *Apis mellifera* | 4 | 72 | 73 | 2 | 2 | 0 |
| 27 | *Apis mellifera* | 7 | 59 | 142 | 2 | 4 | 1 |
| 29 | *Bombus* spp. | 11 | 104 | 254 | 1 | 3 | 1 |
| 30 | *Apis mellifera* | 14 | 54 | 55 | 3 | 4 | 1 |
| 37 | *Bombus* spp. | 126 | 121 | 187 | 1 | 4 | 1 |
| 38 | *Bombus* spp. | 17 | 80 | 104 | 1 | 4 | 0 |
| 59 | *Apis mellifera* | 84 | 62 | 62 | 6 | 7 | 1 |
| 74 | *Bombus* spp. | 10 | 83 | 83 | 1 | 1 | 0 |
| 76 | *Bombus* spp. | 20 | 88 | 132 | 1 | 2 | 0 |
| 79 | *Bombus* spp. | 136 | 57 | 202 | 1 | 2 | 0 |
| 81 | *Bombus* spp. | 47 | 38 | 200 | 1 | 5 | 0 |
| 97 | *Bombus* spp. | 21 | 59 | 90 | 1 | 1 | 1 |
| 100 | *Bombus* spp. | 110 | 57 | 62 | 1 | 1 | 1 |
| 101 | *Bombus* spp. | 39 | 54 | 127 | 2 | 6 | 0 |
| 102 | *Bombus* spp. | 66 | 52 | 172 | 1 | 5 | 1 |
| 109 | *Bombus* spp. | 36 | 73 | 114 | 2 | 4 | 1 |
| 110 | *Apis mellifera* | 22 | 69 | 83 | 3 | 5 | 1 |
| 114 | *Apis mellifera* | 7 | 47 | 226 | 2 | 5 | 1 |
| 136 | *Bombus* spp. | 136 | 102 | 273 | 2 | 5 | 1 |
| 140 | *Bombus* spp. | 55 | 81 | 243 | 1 | 4 | 0 |
| 148 | *Bombus* spp. | 9 | 44 | 118 | 1 | 2 | 0 |
| 149 | *Bombus* spp. | 54 | 50 | 220 | 4 | 4 | 1 |
| 151 | *Bombus* spp. | 16 | 44 | 112 | 1 | 2 | 0 |
| 154 | *Apis mellifera* | 12 | 67 | 176 | 1 | 17 | 1 |
| 160 | *Apis mellifera* | 14 | 51 | 100 | 3 | 12 | 1 |
| 162 | *Apis mellifera* | 21 | 50 | 92 | 1 | 14 | 1 |
| 163 | *Apis mellifera* | 9 | 73 | 193 | 2 | 4 | 1 |
| 210 | *Bombus* spp. | 17 | 118 | 209 | 30 | 37 | 1 |
| 214 | *Bombus* spp. | 104 | 95 | 256 | 25 | 57 | 1 |
| 225 | *Bombus* spp. | 106 | 90 | 106 | 1 | 0 | 1 |
| 231 | *Bombus* spp. | 96 | 72 | 362 | 1 | 0 | 1 |
| 249 | *Apis mellifera* | 6 | 92 | 362 | 1 | 1 | 1 |
| 251 | *Bombus* spp. | 53 | 85 | 268 | 1 | 1 | 1 |
| 261 | *Bombus* spp. | 18 | 77 | 275 | 1 | 3 | 1 |
| 276 | *Apis mellifera* | 32 | 94 | 193 | 20 | 26 | 1 |
| 317 | *Bombus* spp. | 3 | 64 | 212 | 1 | 0 | 1 |
| 323 | *Apis mellifera* | 19 | 62 | 99 | 7 | 12 | 1 |
| 324 | *Bombus* spp. | 31 | 42 | 98 | 13 | 26 | 1 |
| 325 | *Bombus* spp. | 26 | 55 | 101 | 4 | 11 | 1 |
| 326 | *Apis mellifera* | 6 | 53 | 76 | 3 | 13 | 1 |
| 331 | *Bombus* spp. | 46 | 69 | 119 | 1 | 30 | 1 |
| 332 | *Bombus* spp. | 51 | 41 | 67 | 1 | 11 | 1 |
| 333 | *Bombus* spp. | 27 | 52 | 131 | 1 | 6 | 1 |
| 334 | *Apis mellifera* | 4 | 37 | 93 | 3 | 6 | 1 |
| 347 | *Bombus* spp. | 65 | 67 | 270 | 1 | 1 | 0 |
| 357 | *Bombus* spp. | 150 | 84 | 249 | 1 | 2 | 1 |
| 364 | *Apis mellifera* | 13 | 45 | 75 | 5 | 11 | 0 |
| 365 | *Apis mellifera* | 127 | 59 | 77 | 15 | 28 | 1 |
| 366 | *Apis mellifera* | 12 | 75 | 276 | 3 | 27 | 1 |
| 367 | *Apis mellifera* | 26 | 39 | 112 | 2 | 19 | 1 |
| 369 | *Apis mellifera* | 34 | 74 | 103 | 4 | 5 | 1 |
| 370 | *Bombus* spp. | 62 | 119 | 162 | 7 | 11 | 1 |
| 371 | *Apis mellifera* | 27 | 70 | 111 | 2 | 15 | 1 |
| 372 | *Apis mellifera* | 31 | 37 | 94 | 1 | 2 | 1 |
| 373 | *Apis mellifera* | 7 | 34 | 70 | 1 | 1 | 1 |
| 375 | *Apis mellifera* | 44 | 45 | 107 | 1 | 7 | 1 |
| 376 | *Apis mellifera* | 19 | 42 | 119 | 3 | 10 | 1 |
| 377 | *Apis mellifera* | 30 | 37 | 37 | 4 | 13 | 1 |
| 378 | *Apis mellifera* | 24 | 36 | 105 | 1 | 4 | 1 |
| 383 | *Apis mellifera* | 8 | 62 | 62 | 1 | 3 | 1 |
| 384 | *Apis mellifera* | 26 | 43 | 80 | 1 | 4 | 1 |
| 385 | *Apis mellifera* | 8 | 35 | 88 | 1 | 0 | 1 |
| 389 | *Apis mellifera* | 6 | 50 | 138 | 1 | 8 | 1 |
| 390 | *Apis mellifera* | 31 | 57 | 57 | 3 | 7 | 1 |
| 391 | *Apis mellifera* | 10 | 45 | 89 | 2 | 13 | 1 |
| 392 | *Apis mellifera* | 52 | 65 | 77 | 7 | 21 | 1 |
| 393 | *Apis mellifera* | 2 | 55 | 89 | 1 | 11 | 1 |
| 394 | *Apis mellifera* | 24 | 33 | 36 | 2 | 10 | 0 |
| 395 | *Bombus* spp. | 88 | 59 | 59 | 3 | 14 | 1 |
| 396 | *Apis mellifera* | 22 | 41 | 48 | 5 | 13 | 1 |
| 398 | *Apis mellifera* | 39 | 61 | 101 | 4 | 15 | 0 |
| 399 | *Apis mellifera* | 19 | 47 | 70 | 1 | 7 | 1 |
| 400 | *Apis mellifera* | 15 | 53 | 58 | 1 | 13 | 1 |
| 401 | *Apis mellifera* | 9 | 31 | 41 | 4 | 14 | 1 |
| 402 | *Apis mellifera* | 15 | 29 | 36 | 9 | 11 | 1 |

^1^All values of *S_d_* for each focal inflorescence were 0 or 1 because of one or any combination of the following reasons: most inflorescences had a single pollinium inserted (the mode insertion rate was one pollinium), the multiple pollinia inserted into a single inflorescence were either all self or all outcrossed pollinia, and we successfully genotype only one of the pollinia inserted into an inflorescence due to the extremely small amount of genetic material in each pollinium.
