# Supplementary material for: Self-pollination rate and floral-display size in Asclepias syriaca (Common Milkweed) with regard to floral-visitor taxa
Source: BMC Evol Biol. 2014 Jun 23;14:144. doi: 10.1186/1471-2148-14-144 (PMC4080991; doi:10.1186/1471-2148-14-144)
Supplement: Additional file 2: Table S2 — Means (± SE) of the maternal (M) and inserted-pollinium (I) genotypic data for the three visitor taxa across four polymorphic-microsatellite-locus primer sequences. α is the probability of erroneous pollen-source assignment. Round-robin values were calculated using the methodology described in Park and Werth [51]. Alleles per locus, frequency of the most common allele per locus, genotypes per locus, and frequency of the most common genotype per locus are averaged across four polymorphic-microsatellite-locus primer sequences from O’Quinn and Fishbein [48] (Asyr-C4, Asyr-C102, Asyr-C103, Asyr-C109). The overall values are sample-size-weighted means of the values calculated for each visitor taxon. [file 1471-2148-14-144-S2.doc]

**Additional file 2: Table S2 Means (± SE) of the maternal (M) and inserted-pollinium (I) genotypic data for the three visitor taxa across four polymorphic-microsatellite-locus primer sequences.** α is the probability of erroneous pollen-source assignment. Round-robin values were calculated using the methodology described in Park and Werth [51]. Alleles per locus, frequency of the most common allele per locus, genotypes per locus, and frequency of the most common genotype per locus are averaged across four polymorphic-microsatellite-locus primer sequences from O'Quinn and Fishbein [48] (Asyr-C4, Asyr-C102, Asyr-C103, Asyr-C109). The overall values are sample-size-weighted means of the values calculated for each visitor taxon.

|  | *Apis mellifera* | | | | *Bombus* spp. | | | | lepidopterans | | | | overall | | | |
| --- | --- | --- | --- | --- | --- | --- | --- | --- | --- | --- | --- | --- | --- | --- | --- | --- |
|  | Round-robin (SE) | | Unadjusted (SE) | | Round-robin (SE) | | Unadjusted (SE) | | Round-robin (SE) | | Unadjusted (SE) | | Round-robin (SE) | | Unadjusted (SE) | |
|  | M | I | M | I | M | I | M | I | M | I | M | I | M | I | M | I |
| Alleles per locus | 2.1 (0.20) | 1.7 (0.16) | 2.1 (0.20) | 1.7 (0.17) | 2.5 (0.26) | 2.1 (0.28) | 2.5 (0.24) | 2.1 (0.30) | 1.4 (0.12) | 1.6 (0.16) | 1.4 (0.12) | 1.5 (0.16) | 2.2 (0.14) | 1.8 (0.14) | 2.2 (0.14) | 1.8 (0.14) |
| Frequency of most common allele per locus | 0.77 (0.04) | 0.74 (0.04) | 0.76 (0.04) | 0.75 (0.04) | 0.70 (0.06) | 0.70 (0.09) | 0.67 (0.05) | 0.70 (0.09) | 0.84 (0.08) | 0.84 (0.07) | 0.84 (0.07) | 0.83 (0.07) | 0.75 (0.03) | 0.73 (0.04) | 0.73 (0.03) | 0.74 (0.04) |
| Genotypes per locus | 2.0 (0.20) | 1.6 (0.16) | 2.0 (0.20) | 1.6 (0.16) | 2.5 (0.28) | 2.0 (0.31) | 2.5 (0.26) | 2.0 (0.32) | 1.4 (0.12) | 1.6 (0.17) | 1.4 (0.12) | 1.6 (0.16) | 2.2 (0.15) | 1.8 (0.15) | 2.2 (0.14) | 1.7 (0.15) |
| Frequency of most common genotype per locus | 0.51 (0.07) | 0.68 (0.04) | 0.52 (0.07) | 0.68 (0.04) | 0.38 (0.09) | 0.55 (0.14) | 0.37 (0.09) | 0.55 (0.13) | 0.77 (0.06) | 0.78 (0.08) | 0.78 (0.06) | 0.76 (0.07) | 0.49 (0.05) | 0.64 (0.06) | 0.49 (0.05) | 0.64 (0.06) |
| α | – | 0.35 (0.1) | – | 0.32 (0.09) | – | 0.05 (0.12) | – | 0.04 (0.11) | – | 0.74 (0.24) | – | 0.77 (0.28) | – | 0.28 (0.07) | – | 0.26 (0.07) |
